# Supplementary material for: Rapid Detection of Staphylococcus aureus in Milk and Pork via Immunomagnetic Separation and Recombinase Polymerase Amplification
Source: Microbiol Spectr. 2023 Feb 27;11(2):e02249-22. doi: 10.1128/spectrum.02249-22 (PMC10101137; doi:10.1128/spectrum.02249-22)

Supporting Informations

Rapid detection of *Staphylococcus aureus* in milk and pork via immunomagnetic separation and recombinase polymerase amplification

Running title: Rapid detection of *S.aureus* in food by IMBs-RPA

Runan Cheng[a], Lei Li[a], Sihui Zhen[a], Honglei Liu[a], Zhouhui Wu[a], Yu Wang[a], Zhen Wang[a#]

[a] Beijing Key Laboratory of Traditional Chinese Veterinary Medicine, Animal Science and Technology College, Beijing University of Agriculture, Beijing 102206, China

# Corresponding author: Zhen Wang, Email: wangzhen3355@163.com

Table S1. Eight primer pairs for RPA reaction.

Number	Primer Name	Primer sequences(5'-3')	Product Size(bp)
1	*nuc*-1.1-F	CATCACAAACAGGTAACGGCGTAAATAGAA	191
	nuc-1.1-R	TGTTTCAGGTGTATCAACCAATAATAGTCT	
2	*nuc*-1.2-F	TCACAAACAGGTAACGGCGTAAATAGAAGT	183
	nuc-1.2-R	AGGTGTATCAACCAATAATAGTCTGAATGT	
3	*nuc*-2.1-F	CAAACAGATAACGGCGTAAATAGAAGTGGT	130
	nuc -2.1-R	TAATTTAACCGTATCACCATCAATCGCTTTA	
4	*nuc* -2.2-F	ACAAACAGATAACGGCGTAAATAGAAGTGGT	204
	nuc -2.2-R	CCTTTTTTAGGATGCTTTGTTTCAGGTGTAT	
5	*nuc* -2.3-F	CATCACAAACAGATAACGGCGTAAATAGAAG	208
	nuc -2.3-R	CCTTTTTTAGGATGCTTTGTTTCAGGTGTAT	
6	nuc -2.4-F	ACAAACAGATAACGGCGTAAATAGAAGTGGT	188
	nuc -2.4-R	TTGTTTCAGGTGTATCAACCAATAATAGTCT	
7	*nuc* -3-F	CTTATAGGGATGGCTATCAGTAATGTTTCG	153
	nuc -3-R	TCTATTTACGCCATTATCTGTTTGTGATGC	
8	*nuc* -4-F	CCTGCGACATTAATTAAAGCGATTGATGGTGATACG	294
	nuc -4-R	CAAGCCTTGACGAACTAAAGCTTCGTTTAC	

FIG S1 Detection of *S. aureus* in (A) raw milk and (B) chilled pork samples through the IMBs–PCR method. The red arrows indicate the presence of *S. aureus* in the sample.

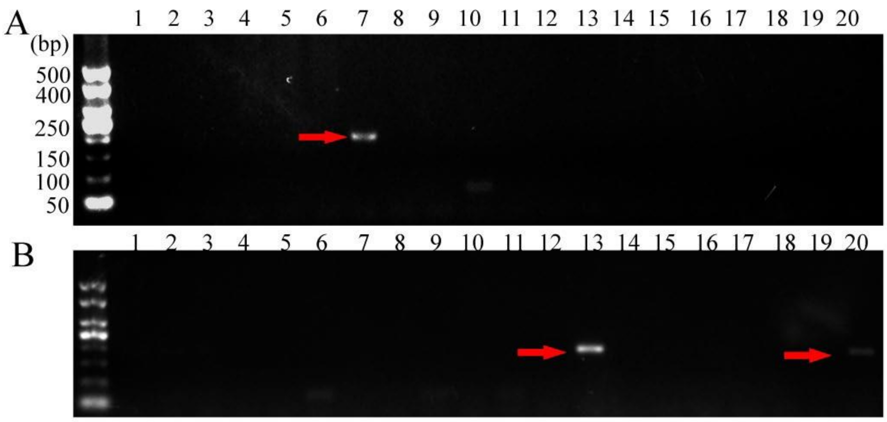

Supplement: Supplemental file 1 — Supplemental material. Download spectrum.02249-22-s0001.pdf, PDF file, 0.1 MB [file spectrum.02249-22-s0001.pdf]
